# Supplementary material for: Trained immunity modulates inflammation-induced fibrosis
Source: Nat Commun. 2019 Dec 11;10:5670. doi: 10.1038/s41467-019-13636-x (PMC6906311; doi:10.1038/s41467-019-13636-x)
Supplement: Supplementary file 1 — Supplementary Information [file 41467_2019_13636_MOESM1_ESM.pdf]

Supplementary information

Trained immunity modulates inflammation-induced fibrosis

By Jeljeli *et al*.

Supplementary Figure 1

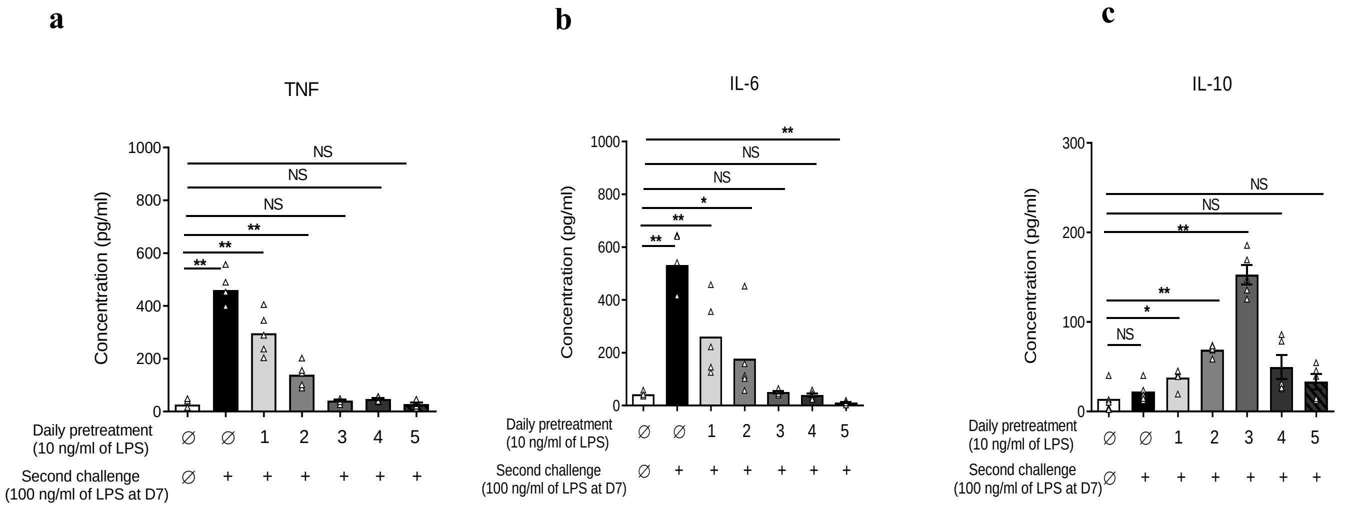

Supplementary Figure 1. Dose-response adjustment for in vitro macrophage LPS training
(a-c) A dose-response adjustment for in vitro macrophage (lipopolysaccharide) LPS training was conducted in order to establish the optimal LPS dose to achieve endotoxin tolerance as reflected by pro-inflammatory cytokines (TNF and IL-6) blockage. Bone marrow derived macrophages were incubated with 10 ng/ml LPS for 1 up to 5 successive days, with daily change of the culture medium and were challenged by 100 ng/ml at day 7. Culture supernatants were collected and stored at -80°C for cytokine assessment. Cytokine production was also evaluated in cells without any LPS challenge (basal secretion) and after a unique inflammatory challenge on untrained cells (inflammatory response). Cell death was measured by trypan blue uptake and light microscopy. Optimal dose and duration of the challenge (10 ng/ml daily for 3 days) were selected on the training scheme that suppresses the pro-inflammatory cytokine production with a preservation of the cell viability (>90% of total cells).
Each box represents mean concentration (pg/ml) ± SEM of the n=5 biologically independent samples. The two-way ANOVA test with Bonferroni correction was used to detect significant differences between the groups. NS: Not significant; *p≤0.05; **p≤ 0.01. Source data are provided as a Source Data file.

Supplementary Figure 2

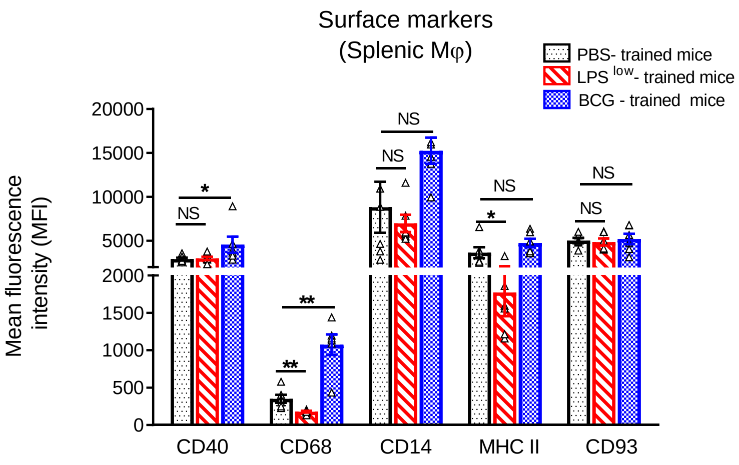

Supplementary Figure 2. Effects of in vivo immune training on splenic macrophage phenotype
Flow cytometric analysis of splenic Mφ (Macrophages) phenotype at day 7 after training. (Remaining assessed macrophages markers). Data represent the mean fluorescence index (MFI) and SEM from n=6 biologically independent mice samples. The ANOVA test with Bonferroni correction was used to detect significant differences between the groups. NS: Non significant; *p≤0.05; **p≤ 0.01; ***p≤0.001. Source data are provided as a Source Data file.

Supplementary Figure 3

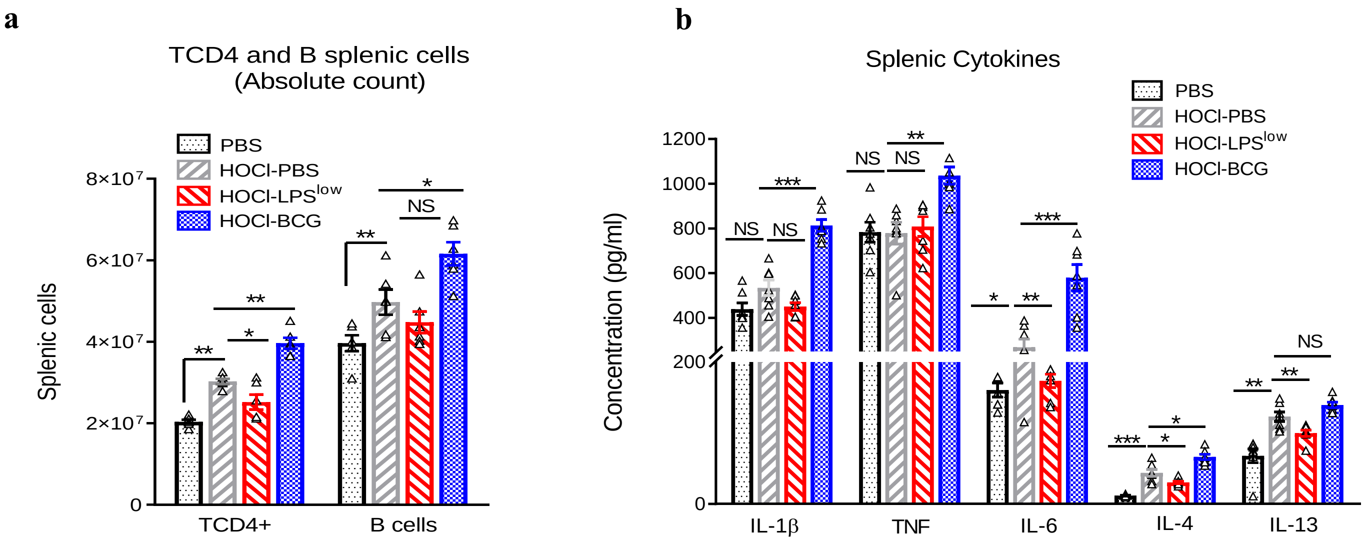

Supplementary Figure 3. In vivo immune training impacts cytokine capacity production and T cells absolute count in murine HOCl-induced SSc during the inflammatory phase of the disease
(a) Flow cytometric characterization of splenic B cells and CD4+ T cells at day 22. Data represent the absolute count for T CD4+ and B cells with SEM from n=5 biologically independent samples. **(b)** Cytokine production in spleen cells culture supernatant (ELISA assessment) after stimulation with Concanavaline A. Each box represents mean ± SEM obtained with cell culture from n=7 biologically independent mice. The ANOVA test with Bonferroni correction was used to detect significant differences between the groups. NS: Not significant; *p≤0.05; **p≤ 0.01; ***p≤0.001. Source data are provided as a Source Data file.

Supplementary Figure 4

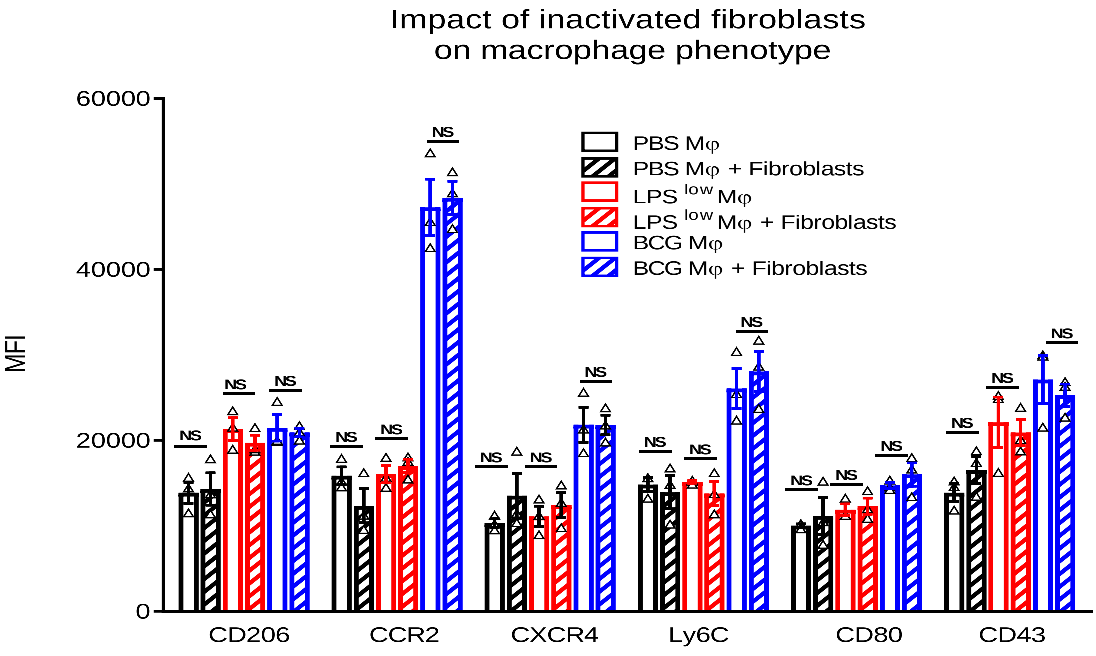

Supplementary Figure 4. PBS (non-activated) fibroblasts do not affect macrophage phenotype
Macrophage phenotype was assessed by flow cytometry after 48h of co-culture with non-activated fibroblasts. Data represent the mean fluorescence index (MFI) and SEM from n=3 biologically independent samples of each condition. The ANOVA test with Bonferroni correction was used to detect significant differences between the groups. NS: Non-significant. Mφ: Macrophages. Source data are provided as a Source Data file.

Supplementary Figure 5

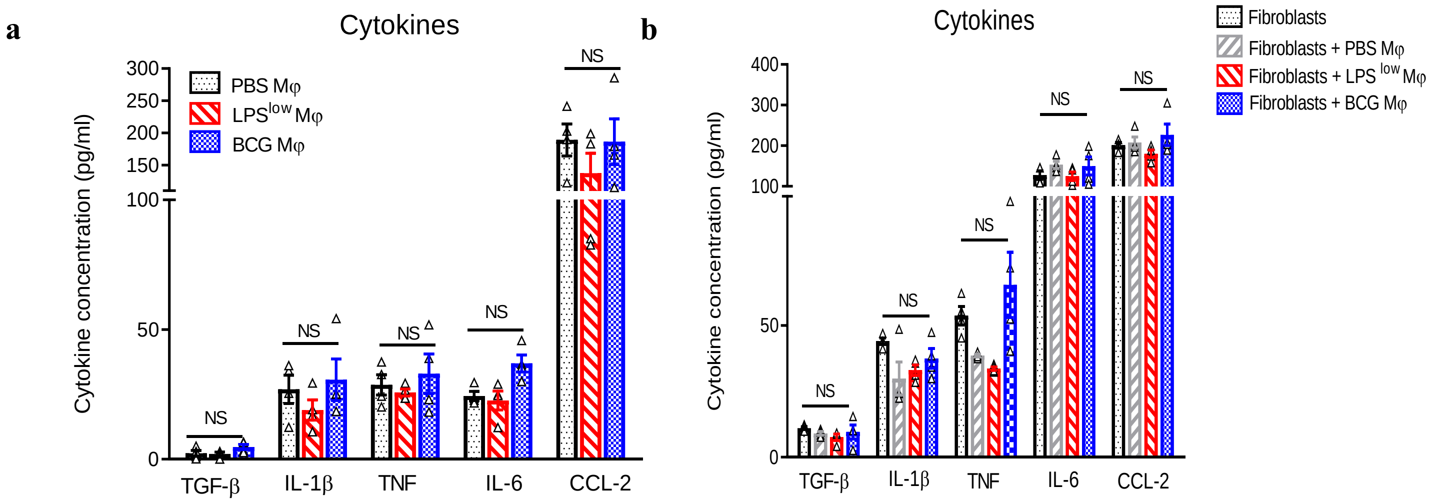

Supplementary Figure 5. Cytokine production of trained macrophages cultured alone and with non-activated fibroblasts

(a) Basal cytokine production of un-stimulated murine trained macrophage cultured alone in medium. Each box represents mean ± SEM from n=4 biologically independent samples. **(b)** Cytokine production of murine trained macrophage after 48h of co-culture with non-activated fibroblasts. Each box represents mean ± SEM from n=4 biologically independent samples. The ANOVA test with Bonferroni correction was used to detect significant differences between the groups. NS: Not significant. Mφ: Macrophages. Source data are provided as a Source Data file.

Supplementary Figure 6

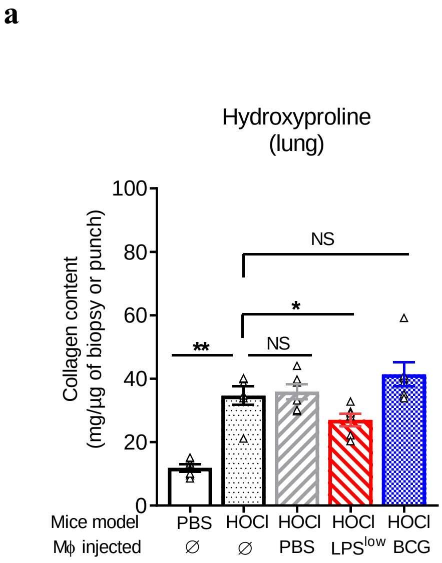

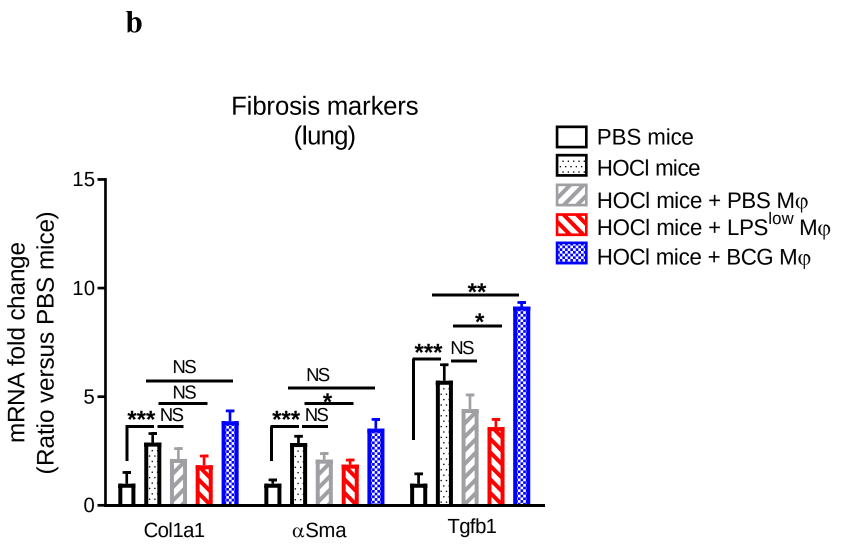

Supplementary Figure 6. Impact of adoptive transfer of trained macrophages on pulmonary fibrosis

(a) Collagen content in lung (Hydroxyproline dosage, mg/lobe of biopsy). Each box represents mean ± SEM from n=6 biologically independent mice. The ANOVA test with Bonferroni correction was used to detect significant differences between the groups. NS: Not significant; *p≤0.05; **p≤ 0.01; ***p≤0.001 **(b)** *Col1a1*, *α-sma* and *tgfb1* mRNA levels in lung. Results are expressed as fold increase ± SEM versus the control group PBS derived from n=6 biologically independent mice. NS: Not significant; *p≤0.05; **p≤ 0.01; ***p≤0.001, (Unpaired t-test). Mφ: Macrophages. Source data are provided as a Source Data file.

Supplementary Figure 7

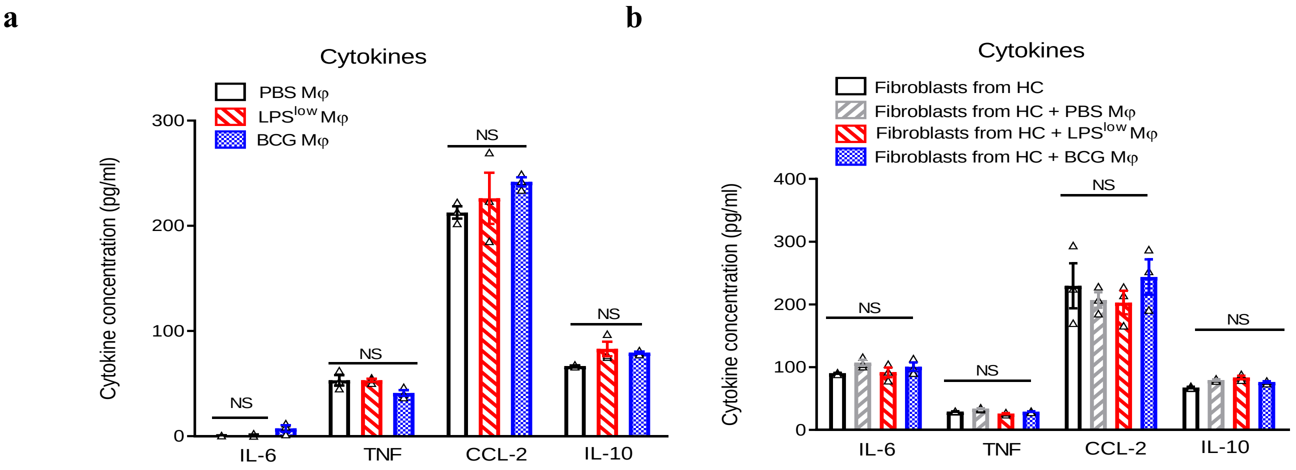

Supplementary Figure 7. Basal cytokine production of trained macrophages cultured alone and with fibroblasts from healthy control

(a) Basal cytokine production of un-stimulated human trained macrophages cultured alone in medium. **(b)** Cytokine production of human trained macrophage after 48h of co-culture with healthy fibroblasts. Each box represents mean ± SEM from n=3 biologically independent samples. The ANOVA test with Bonferroni correction was used to detect significant differences between the groups. Mφ: Macrophages. NS: Not significant; *$p \leq 0.05$; **$p \leq 0.01$; ***$p \leq 0.001$**.** Source data are provided as a Source Data file.

Supplementary Figure 8

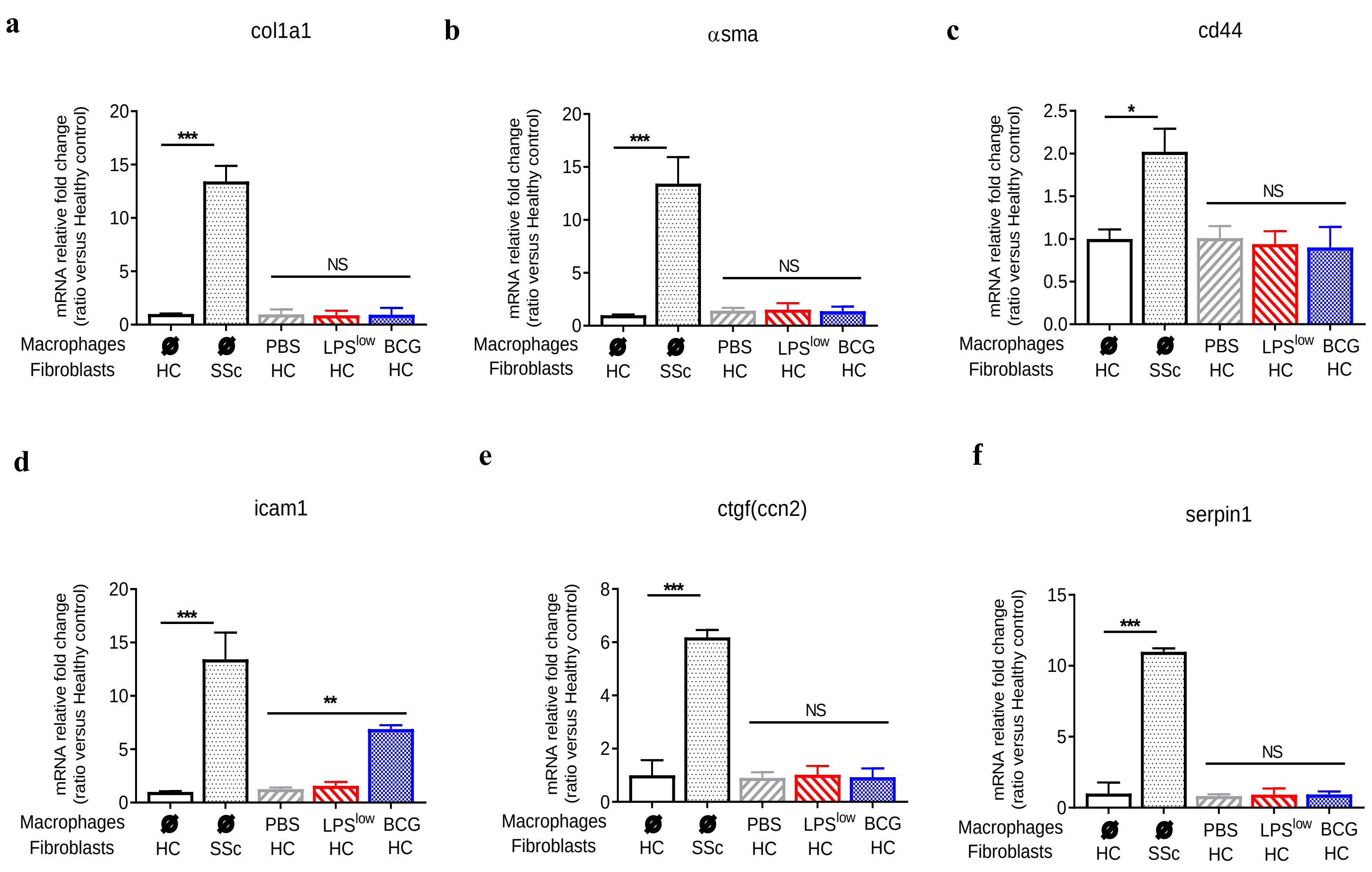

Supplementary Figure 8. Gene expression of fibroblasts from HC co-cultured with trained macrophages

(a-f) RT-qPCR assessment of *α-sma*, *col1a1*, *cd44*, *icam-1*, *ctgf* and *serpin1* mRNA levels in fibroblasts from healthy controls after 48h of co-culture with trained macrophages and results are expressed as mean fold increase ± SEM from n=3 biologically independent samples. mRNA expression from diseased SSc fibroblasts is shown in each graph as a positive control. NS: Not significant; *p≤0.05; **p≤ 0.01; ***p≤0.001, (Unpaired t-test). Source data are provided as a Source Data file.

Supplementary Figure 9

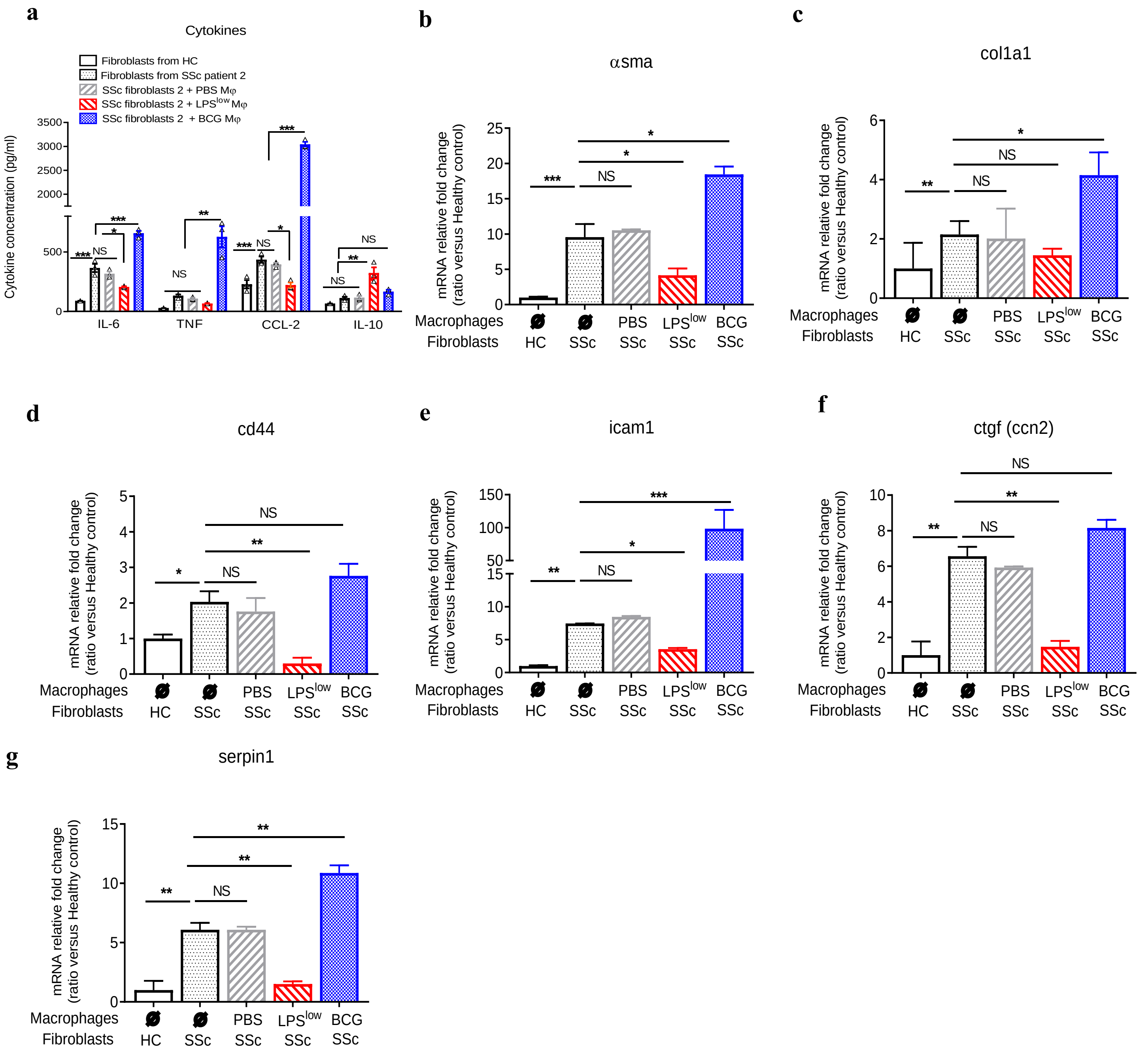

Supplementary Figure 9. Additional experiment conducted on another myofibroblasts lineage from a second SSc patient.

(a) Cytokine production (IL-6, TNF, CCL-2 and IL-10) was assessed by ELISA in co-culture supernatant after 48 h of incubation (pg/mL). Each box represents mean ± SEM from n=3 biologically independent samples. The ANOVA test with Bonferroni correction was used to detect significant differences between the groups. **(b-g)** RT-qPCR assessment of *α-sma*, *col1a1*, *cd44*, *icam-1*, *ctgf* and *serpin1* mRNA levels, after 48h of co-culture and results are expressed as mean fold increase ± SEM from n=3 biologically independent samples. NS: Not significant; *p≤0.05; **p≤ 0.01; ***p≤0.001 (Unpaired t-test). Mφ: Macrophages. Source data are provided as a Source Data file.

Supplementary Figure 10

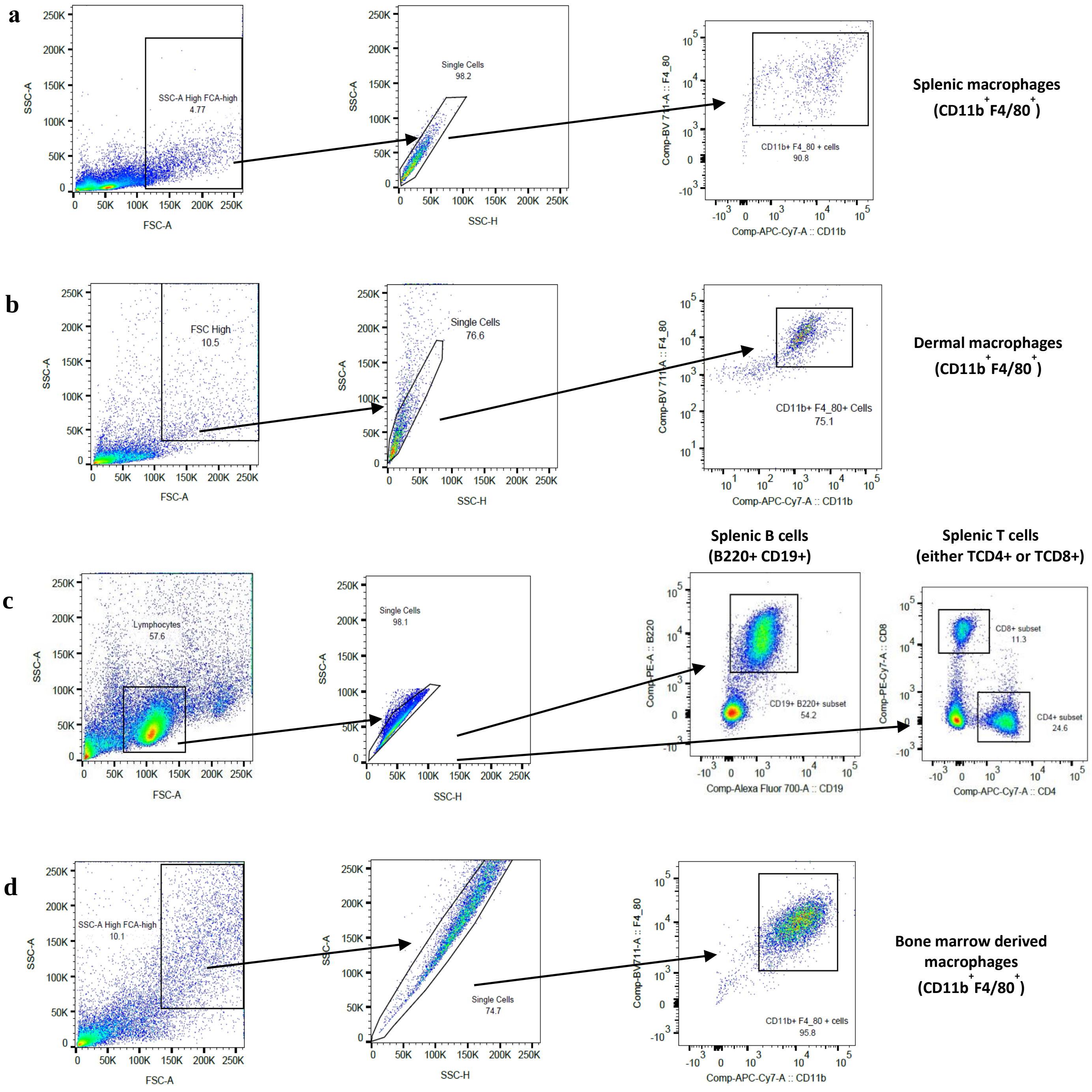

Supplementary Figure 10. Gating strategies used for flow cytometry analysis

(a) Flow cytometric gating strategy to detect splenic macrophages from Balb/c mice for further analysis of surface markers expression. High FCS-A population was considered and splenic macrophages were identified as $CD11b^{+}$ $F4/80^{+}$ double positive cells and presented in Figs.1b-g and Supplementary Fig.2. **(b)** Flow cytometric gating strategy to identify dermal macrophages from Balb/c mice for further surface markers analysis present on Figs.2b and 5g. **(c)** Flow cytometric gating strategy to analyze the frequency and expression of activation markers on B cells ($B220^{+}$ $CD19^{+}$) and $TCD4^{+}$ cells ($CD4^{+}$ $CD8^{-}$) isolated from spleen, present on Fig.2b and Supplementary Fig.3b **(d)** Flow cytometric gating strategy to identify macrophages (Gated as $CD11b^{+}$ $F4/80^{+}$) derived from the bone marrow of Balb/c mice and used for co-culture experiments illustrated on Fig.4b and Supplementary Fig. 4.

Supplementary Table 1: List of primers sequences used for real time quantitative PCR and ChiP analysis

Murine primers used for ChiP analysis		
**Gene Promoter**	**Forward sequence 5'-3'**	**Reverse sequence 5'-3'**
Tnf	CACCCCGAAGTTCAGTAGACA	GAACTGGCAGAAGAGGCACT
il-6	TTTCTCCACGCAGGAGACTT	TCCACGATTTCCCAGAGAAC
il-10	GTGGCTATCACCGTGCAGTA	AAGCAACTGCCTCTCTGAGC
**Murine primers used for RT-qPCR analysis for tissues and cells**		
**Gene**	**Forward sequence 5'-3'**	**Reverse sequence 5'-3'**
β-actin	ACCACCATGTACCCAGGCATT	CCACACAGAGTACTTGCGCTCA
α-sma	CTACGAACTGCCTGACGGG	GCTGTTATAGGTGGTTTCGTGG
Col1a1	TGTTCGTGGTTCTCAGGGTAG	TTGTCGTAGCAGGGTTCTTTC
Il-13	GCAGCATGGTATGGAGTGTG	TGGCGAAACAGTTGCTTTGT
Tgfb1	CACCGGAGAGCCCTGGATA	TGTACAGCTGCCGCACACA
**Human primers used for RT-qPCR analysis**		
**Gene**	**Forward sequence 5'-3'**	**Reverse sequence 5'-3'**
Gapdh	GCCACATCGCTCAGACAC	GCCCAATACGACCAAATCC
α-sma	CCACCGCAAATGCTTCTAAGT	GGCAGGAATGATTTGGAAAGG
Col I	GAACGCGTGTCATCCCTTGT	GAACGAGGTAGTCTTTCAGCAACA
Cd44	CGGACACCATGGACAAGTTT	GAAAGCCTTGCAGAGGTCAG
icam-1	CAGAGGTTGAACCCCACAGT	CCTCTGGCTTCGTCAGAATC
Ctgf (ccn2)	CAGCATGGACGTTCGTCTG	AACCACGGTTTGGTCCTTGG
Serpin1	AGTGGACTTTTCAGAGGTGGA	GCCGTTGAAGTAGAGGGCATT
