# Supplementary material for: Trained immunity modulates inflammation-induced fibrosis
Source: Nat Commun. 2019 Dec 11;10:5670. doi: 10.1038/s41467-019-13636-x (PMC6906311; doi:10.1038/s41467-019-13636-x)
Supplement: Supplementary file 3 — Reporting Summary [file 41467_2019_13636_MOESM3_ESM.pdf]

nature research

Corresponding author(s): DBPR NCOMMS-19-13768B

Last updated by author(s): Oct 10, 2019

Reporting Summary

Nature Research wishes to improve the reproducibility of the work that we publish. This form provides structure for consistency and transparency in reporting. For further information on Nature Research policies, see Authors & Referees and the Editorial Policy Checklist .

Statistics

For all statistical analyses, confirm that the following items are present in the figure legend, table legend, main text, or Methods section.

n/a	Confirmed	
☐	☒	The exact sample size (*n*) for each experimental group/condition, given as a discrete number and unit of measurement
☐	☒	A statement on whether measurements were taken from distinct samples or whether the same sample was measured repeatedly
☐	☒	The statistical test(s) used AND whether they are one- or two-sided<br>*Only common tests should be described solely by name; describe more complex techniques in the Methods section.*
☒	☐	A description of all covariates tested
☐	☒	A description of any assumptions or corrections, such as tests of normality and adjustment for multiple comparisons
☐	☒	A full description of the statistical parameters including central tendency (e.g. means) or other basic estimates (e.g. regression coefficient) AND variation (e.g. standard deviation) or associated estimates of uncertainty (e.g. confidence intervals)
☐	☒	For null hypothesis testing, the test statistic (e.g. *F*, *t*, *r*) with confidence intervals, effect sizes, degrees of freedom and *P* value noted<br>*Give P values as exact values whenever suitable.*
☒	☐	For Bayesian analysis, information on the choice of priors and Markov chain Monte Carlo settings
☒	☐	For hierarchical and complex designs, identification of the appropriate level for tests and full reporting of outcomes
☒	☐	Estimates of effect sizes (e.g. Cohen's *d*, Pearson's *r*), indicating how they were calculated

Our web collection on statistics for biologists contains articles on many of the points above.

Software and code

Policy information about availability of computer code

Data collection	No software was used for data collection.
Data analysis	GraphPad Prism 8.0 for data analysis.

For manuscripts utilizing custom algorithms or software that are central to the research but not yet described in published literature, software must be made available to editors/reviewers. We strongly encourage code deposition in a community repository (e.g. GitHub). See the Nature Research guidelines for submitting code & software for further information.

Data

Policy information about availability of data

All manuscripts must include a data availability statement. This statement should provide the following information, where applicable:

- Accession codes, unique identifiers, or web links for publicly available datasets
- A list of figures that have associated raw data
- A description of any restrictions on data availability

All data generated or analyzed during this study are included in the Source Data file. For further request please contact the corresponding author.

Field-specific reporting

Please select the one below that is the best fit for your research. If you are not sure, read the appropriate sections before making your selection.

☒ Life sciences ☐ Behavioural & social sciences ☐ Ecological, evolutionary & environmental sciences

For a reference copy of the document with all sections, see nature.com/documents/nr-reporting-summary-flat.pdf

Life sciences study design

All studies must disclose on these points even when the disclosure is negative.

Sample size	The sample size calculation for this study was based on an online calculator Ref#Rosner B. Fundamentals of Biostatistics. 7th ed. Boston, MA: Brooks/Cole; 2011 to detect at least 66% difference between the groups with an intra group variance of 5%. The probability of a type-I error (α) - finding a difference when a difference does not exist - was set to 5%. The probability of a type-II error (β) -- not detecting a difference when one actually exists-- was set to 20%.
Data exclusions	No data was excluded from the analysis.
Replication	The findings of the macrophage training of the first experiment were verified 3 times successfully with reproducible results. The reproduction has permitted to extend also the panel of markers explored. As Results of macrophage training (Cytokine production and markers expression) were reproduced in the experiment of figure 2 (with the similar tendency of macrophage training in Figure 1) and as the experiment from Figure 2 was pricey and time consuming, its entire replication couldn't be done.<br>Findings of the co-culture were verified and reproduced from Figure 4.<br>Systemic sclerosis induction and macrophage infusion (Figure 5) has shown the same training profile as in the experiments #1 and #2, given the complexity of Bone marrow macrophage extraction and training to adjust the scheduling with the weekly infusion of the macrophages, replication of this experiment couldn't be performed entirely.<br>Co-culture of trained macrophages and fibroblast form patients (Figure 6) were performed twice successfully on two independent patients and macrophages from different donors with the same results.
Randomization	Allocations of the samples of all the experiments were totally randomized.
Blinding	Investigators were blinded during skin fold measurement in all the experiments, samples collection at mice sacrifice and during data analysis.

Reporting for specific materials, systems and methods

We require information from authors about some types of materials, experimental systems and methods used in many studies. Here, indicate whether each material, system or method listed is relevant to your study. If you are not sure if a list item applies to your research, read the appropriate section before selecting a response.

Materials & experimental systems

n/a	Involved in the study	
☐	☒	Antibodies
☒	☐	Eukaryotic cell lines
☒	☐	Palaeontology
☐	☒	Animals and other organisms
☐	☒	Human research participants
☒	☐	Clinical data

Methods

n/a	Involved in the study	
☒	☐	ChIP-seq
☐	☒	Flow cytometry
☒	☐	MRI-based neuroimaging

Antibodies

Antibodies used	F4/80-BV711 (BM8, Catalog#123147, dilution 1/200), CD11b-APC/Cy7 (M1/70, Catalog#101226, dilution 1/200), CD14-PE/Cy7 (Sa14-2, Catalog#123315, dilution 1/100), CD40-FITC (3/23, Catalog#124607, dilution 1/100), CD44-APC (IM7, Catalog#103012, dilution 1/200), CD69-PercP/Cy5.5 (H1.2F3, Catalog#104522, dilution 1/100), CD93-PercP/Cy5.5 (AA4.1, Catalog#136511, dilution 1/100), CXCR4-BV421 (L276F12, Catalog#146511, dilution 1/200), CD282-PE Toll-Like receptor 2 (TLR-2) (CB225, Catalog#148603, dilution 1/200), CD284-PE (TLR-4) (SA15-21, Catalog#145403, dilution 1/200), Ly6-C-PercP/Cy5.5 (HK1.4, Catalog#128011, dilution 1/200), CD275-PE (inducible T-cell COStimulator Ligand ; iCOSL) (HK5.3, Catalog#107405, dilution 1/100), CCR2-PE (SA203G11, Catalog#150609, dilution 1/200), CD3-PE (145-2C11, Catalog#100308, dilution 1/200), CD4-APC-Cy7 (GK1.5 Catalog#100414, dilution 1/200), CD8-PE-Cy7 (53-6.7, Catalog#100722, dilution 1/200) , CD19-Alexa Fluor 700 (6D5, Catalog#115528, dilution 1/150), CD45R/B220-PE (RA3-6B2, Catalog#103208, dilution 1/200) and CD80-APC (16-10A1, Catalog#104714, dilution 1/200) from BioLegend (Ozyme France, 78180 Montigny-le-Bretonneux) and CD45- FITC (RA3-6B2, Catalog#11-0452-82, dilution 1/200), CD11b-BV510 (M1/70, Catalog#562950, dilution 1/200), CD80-FITC (16-10A1, Catalog#553768, dilution 1/200), CD209-APC (DC-SIGN) (LWC06, Catalog#17-2092-80, dilution 1/200), CD43-BV421 (S7, Catalog#562958, dilution 1/200), CD206-Alexa Fluor 647 (MR5D3, Catalog#565250, dilution 1/200), IA-IE-FITC (MHC-II) (2G9, Catalog#553623, dilution 1/100) from eBiosciences (Thermo Fisher Scientific, Villebon-Sur-Yvette, France).
Validation	Each primary antibody has been validated by the manufacturer as BioLegend and eBiosciences (Thermo Fisher Sceintific) provide an extensive library of publications citing their products. The validation is stated in the manufacturers' websites.

Animals and other organisms

Policy information about studies involving animals; ARRIVE guidelines recommended for reporting animal research

Laboratory animals	In this study, six-week-old female BALB/c mice weighing 16-20g were purchased from Janvier Laboratory (Le Genest Saint Isle, France). All mice were housed in ventilated cages with sterile food and water ad libitum throughout the study. Animals received humane care in compliance with the guidelines implemented at our institution (INSERM and University Paris Descartes).
Wild animals	No wild animals were used in this study.
Field-collected samples	This study did not involve field-collected samples.
Ethics oversight	The protocols used in this study were reviewed and approved by the local Ethic committee and registred as Protocol N° CEEA34.CN.023.11.

Note that full information on the approval of the study protocol must also be provided in the manuscript.

Human research participants

Policy information about studies involving human research participants

Population characteristics	Blood samples used for macrophage isolation were obtained from healthy volunteers who gave their informed consent and were aged from 32 to 45 years-old. Human diseased fibroblasts were obtained from 4 millimeters dermal biopsies from the forearm skin with active fibrotic lesions from a 53-years old and a 48 year-old patients with a diffuse form of systemic sclerosis diagnosed in 2017 and 2013 respectively, fulfilling the ACR criteria for SSc and without ongoing immunosuppressive treatment.
Recruitment	No potential of self selection of other biases were presented in this study as Skin biopsies derived from 2 SSc patients and 2 healthy subjects were kindly provided by Pr. Yannick Allanore, Rheumatology Department, Cochin Hospital, Paris, France.
Ethics oversight	The local ethical committee of Cochin Hospital and the local institutional review boards [CCP (Comité de Protection des Personnes) Paris Ile De France3] approved the study, and all subjects provided written informed consent.

Note that full information on the approval of the study protocol must also be provided in the manuscript.

Flow Cytometry

Plots

Confirm that:

- [x] The axis labels state the marker and fluorochrome used (e.g. CD4-FITC).
- [x] The axis scales are clearly visible. Include numbers along axes only for bottom left plot of group (a 'group' is an analysis of identical markers).
- [x] All plots are contour plots with outliers or pseudocolor plots.
- [x] A numerical value for number of cells or percentage (with statistics) is provided.

Methodology

Sample preparation	Spleen cells suspensions were prepared after hypotonic lysis of erythrocytes in potassium acetate solution and three washes in complete RPMI medium. For each mouse, splenocytes were enumerated using a Malassez counting chamber.<br>Skin-derived immune cells isolation was achieved by collecting 8 mm-calibrated dermal punches of tissue were from the shaved back of each mouse and minced into small pieces. Tissue was then placed in a 60mm petri dish containing 1ml of dispase solution (Gibco, ville, pays) with 9 ml of complete RPMI medium and 1mg/ml of collagenase (Sigma-Aldrich Ref C2674-1G) and incubated for 3h at 37 °C in an atmosphere of 5 % CO2. Cell suspension was then filtered in a 70µm cell strainer, washed in complete RPMI medium and counted with Malassez counting chamber. These cell suspensions were used for cytometry analysis after staining with the appropriate antibodies.
Instrument	FACS Fortessa II flow cytometer (BD Biosciences)
Software	FlowJo V10.0
Cell population abundance	After identification, splenic macrophages were typically around 5% of total splenic cells. Dermal macrophages were around 6% of the total dermal cells. Selection of splenic macrophages for in vitro stimulation and cytokine assessment was performed using FACS sorting to generate F4/80 positive cells. The purity of the cells was checked by FACS analysis on randomized samples and exceeded 95 % of CD11b and F4/80 double positive cells.
Gating strategy	High FCS-A population was considered to focus on tissue macrophages. Signal overlap was corrected mathematically by single fluorochrome compensations. Splenic macrophages were identified as CD11b+ F4/80+ double positive cells. Boundaries between positive and negative staining cell populations were defined at 103 of axis scale at least, for BV711 and APC-Cy7 (macrophage identification). For dermal and bone marrow derived macrophages identification, FSC high population was considered in order to

eliminate debris. Purity of the cells was assessed by gating on F4/80 and CD11b double positive cell population. Splenic Lymphocytes were identified as a population between 50 and 150 K of forward scatter and 20 to 100K of side scatter. The CD45RA/B220 and CD19 markers were used to isolate B cell population, while CD4 and CD8 markers were used to identify T cell subpopulations. The Plateforme of Cytometry and Immuno-biology CYBIO of Cochin Institute, Paris known for its expertise in performing flow cytometry and data analysis validated and assisted all the experiments.

☒ Tick this box to confirm that a figure exemplifying the gating strategy is provided in the Supplementary Information.
